# Why different sugarcane cultivars show different resistant abilities to smut? Comparisons of endophytic microbial compositions and metabolic functions in stems of sugarcane cultivars with different abilities to resist smut

**DOI:** 10.1186/s12870-023-04446-x

**Published:** 2023-09-14

**Authors:** Siyu Chen, Zhongliang Chen, Xinru Lin, Xinyan Zhou, Shangdong Yang, Hongwei Tan

**Affiliations:** 1 Guangxi Key Laboratory of Agro-Environment and Agro-Product Safety, National Demonstration Center for Experimental Plant Science Education, Agricultural College, Guangxi University, 100 University Road, Nanning, Guangxi 530004 P.R. China; 2 Guangxi Key Laboratory of Sugarcane Genetic Improvement, Guangxi Academy of Agricultural Sciences, 530007 Guangxi, P.R. China

**Keywords:** Sugarcane (*Saccharum officinarum* L.), Smut, Endophytic, Microbial, Metabolome

## Abstract

To elucidate the mechanisms underlying the resistance to smut of different sugarcane cultivars, endophytic bacterial and fungal compositions, functions and metabolites in the stems of the sugarcane cultivars were analyzed using high-throughput sequencing techniques and nontargeted metabolomics. The results showed that the levels of ethylene, salicylic acid and jasmonic acid in sugarcane varieties that were not sensitive to smut were all higher than those in sensitive sugarcane varieties. Moreover, endophytic fungi, such as *Ramichloridium*, *Alternaria*, *Sarocladium*, *Epicoccum*, and *Exophiala* species, could be considered antagonistic to sugarcane smut. Additionally, the highly active arginine and proline metabolism, pentose phosphate pathway, phenylpropanoid biosynthesis, and tyrosine metabolism in sugarcane varieties that were not sensitive to smut indicated that these pathways contribute to resistance to smut. All of the above results suggested that the relatively highly abundant antagonistic microbes and highly active metabolic functions of endophytes in non-smut-sensitive sugarcane cultivars were important for their relatively high resistance to smut.

## Introduction

Sugarcane smut is caused by *Sporisorium scitamineum*. It was first observed [1] in Natal, South Africa, in 1877. In 2007, smut became a global disease, affecting all sugarcane-producing countries and regions worldwide [2]. Sugarcane smut leads to systemic infection of the stem and changes in stem growth, leading to the production of cysts or whips [3]. Moreover, this fungus produces billions of winter spores, which affect plant tissues.

Sugarcane smut can spread rapidly through spores carried in the atmosphere [4]. In particular, susceptible crops that are severely infected are prone to produce many winter spores, which places high infection pressure on surrounding crops and is one of the important factors affecting the disease level in sugarcane planting areas [5]. Severe smut infection usually not only reduces the sugar recovery rate [6] but also decreases the purity and quality index of sucrose, leading to serious economic losses [7]. As resistant cane varieties could minimize the infectivity of pathogens [8], using resistant varieties is the most effective way to control diseases [9]. Studies have shown that cultivars sensitive to head smut are more likely to produce large numbers of winter spores than insensitive cultivars and produce the spores earlier, and glycosides in bud scales are also related to the smut resistance of some varieties [10].

Most plants are colonized by endophytes [11, 12], and interactions between plants and microorganisms involve complex metabolic pathways, resulting in unique characteristics of endophytes [13]. Plant-related microorganisms can provide valuable nutrients or protective metabolites for the host, improving its adaptive advantage against pathogens [14]. For example, endophytic microorganisms can produce antibiotics, plant hormones, iron carriers, and solubilize phosphorus to promote plant growth [15, 16]. Additionally, endophytes can be used as biological control agents for systemic plant diseases Cook, 1993 [17]. Jayakumar et al. [18] and Gao et al. [19] found that endophytic microorganisms can be used as biological control agents for preventing smut. Among them, endophytic fungi can reduce the risk of smut infection [20, 21]. In addition, endophytes can also promote plant growth by regulating plant hormones or assisting plants in resisting external factors such as pathogens [22, 23]. The plant hormone signaling pathways associated with auxin, abscisic acid, salicylic acid and ethylene-related genes are relatively highly sensitive to smut [24]. Meanwhile, changes in microbial composition also lead to changes in microbial metabolic functions [25, 26]. For example, smut-resistant cane varieties were found to contain higher levels of total and free phenols and lower levels of total sugars and free amino acids [27].

Despite the long-standing and growing interest in the use of fungal and bacterial antagonists and in combating infectious plant diseases, little research has been conducted thus far on the nonchemical control of sugarcane smut [28]. Therefore, in this study, the differences in endophytic microbial communities, metabolites and metabolic functions in the stems of different smut-resistant sugarcane cultivars were analyzed., i.e., (1) differences in endophytic fungal and bacterial community structures among different sugarcane cultivars susceptible to smut and (2) characteristics of metabolites and metabolic function of endophytic microorganisms in different smut-resistant sugarcane cultivars. This study will help elucidate the role of changes in endophytes and their metabolic functions in the smut susceptibility of different sugarcane varieties.

## Material and methods

### Field site description and experimental designs

**Fig. 1:**
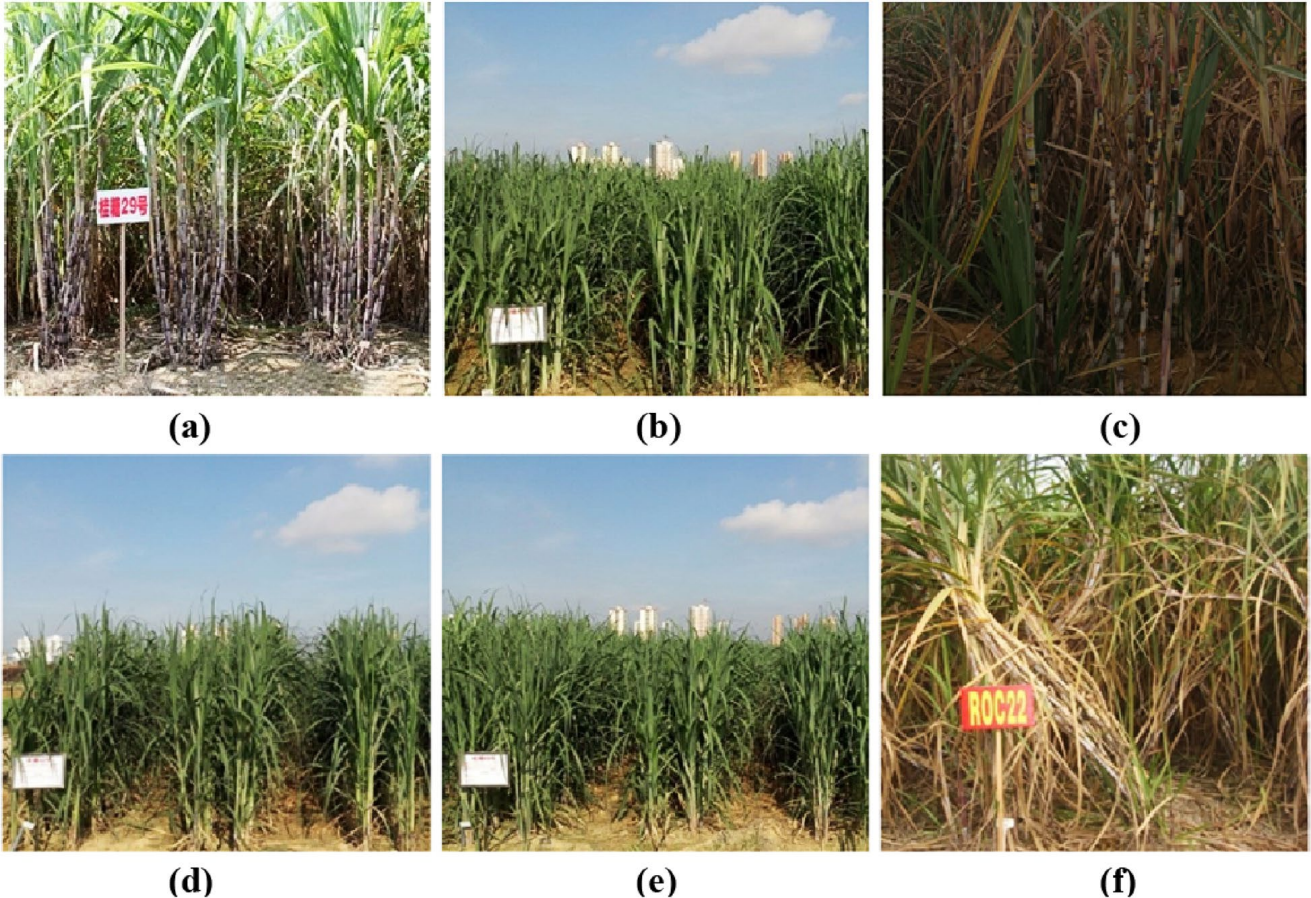
Appearance and morphological characteristics of different susceptible sugarcane cultivars

The experiment was conducted at the Experimental Base of the Sugarcane Research Institute, Guangxi Academy of Agricultural Sciences, Longan County (107°598″E and 23°637″N), Guangxi, China. Three insensitive sugarcane cultivars, GT29 (a), GT43 (b) and GT52 (c), and three smut-susceptible sugarcane cultivars, GT42 (d), GT49 (e) and ROC22 (f), were used in this paper for analysis (Fig. 1). Each sugarcane cultivar was examined in twenty-five repeats. All the seedlings above were grown and managed under identical conditions.

Samples were collected after the sugarcane plants entered the elongation period, and 6 plant samples with consistent growth were randomly collected and mixed into biological replicates. Each variety was examined in triplicate. The collected samples were placed in a sealed sterile bag, labeled and sent back to the laboratory. A soft brush was used to rinse and wipe the stem samples with sterile water for 2 min to remove impurities on the surface of the stems, and then the samples were washed with 75% ethanol for 1 min and 1% NaClO solution for 3 min. Finally, all stems were washed with sterile water for 0.5 min, dried using sterile paper and stored in a -80 °C freezer until DNA extraction.

Moreover, the soil properties at the experimental site were as follows: pH 5.68, organic matter content 8.92 g·kg^−1^, total nitrogen content 0.55 g·kg^−1^, total phosphorus content 0.67 g·kg^−1^, and total potassium content 7.51 g·kg^−1^. The levels of alkaline dissolved nitrogen, available phosphorus and potassium were 15.27 mg·kg^−1^, 0.67 mg·kg^−1^, and 82.8 mg·kg^−1^, respectively.

### Test methods

#### Analysis of endophytic microbial diversity

Total DNA extraction from the stem samples followed by PCR amplification and sequence determination was performed by Shanghai Majorbio Biopharm Technology Co., Ltd. High-throughput sequencing was performed using the MiSeq platform.

Total DNA extraction was performed according to the instructions of the FastDNA® Spin Kit for Endophytic (MP Biomedicals, U.S.), and DNA concentration and purity were measured using a NanoDrop 2000 spectrophotometer (Thermo Fisher Scientific, U.S.). PCR amplification was performed on an ABI GeneAmp® 9700 with the specific primers and sequence types shown in Table 1.

**Table 1:** Sequence type and primer sequences

Sequence type	Primer name	Primer sequence	Length	Sequencing platform
Bacterial 16SrRNA	799F	5′-AACMGGATTAGATACCCKG-3′	593 bp	MiseqPE250
	1192R	5′-ACGGGCGGTGTGTRC-3′		
	799F	5′-AACMGGATTAGATACCCKG-3′	394 bp	
	1193R	5′-ACGTCATCCCCACCTTCC-3′		
ITS	ITS1F	5′-CTTGGTCATTTAGAGGAAGTAA-3′	350 bp	MiSeq PE300
	ITS2F	5′-GCTGCGTTCTTCATCGATGC-3′		

Illumina MiSeq sequencing was performed as follows: PCR products from the same sample were purified using the AxyPrep DNA Gel Extraction Kit (Axygen Biosciences, Union City, CA, USA) and mixed, followed by detection on and recovery from a 2% agarose gel. The recovered products were quantified using a Quantus™ Fluorometer (Promega, USA). Library construction was carried out using the NEXTFLEX® Rapid DNA-Seq Kit.

The PCR amplification process for the 16S rRNA gene was as follows: initial denaturation at 95 °C for 3 min, followed by three cycles of denaturation at 95 °C for 30 s, annealing at 55 °C for 30 s, and extension at 72 °C for 45 s, a single extension at 72 °C for 10 min, and termination at 4 °C. DNA gel extraction kits from AXY (Axygen Biosciences, Union City, California, USA) were used according to the manufacturer’s instructions to extract and purify PCR products from a 2% agarose gel and quantify them by a quantum fluorimeter (Promega, USA). Sequence data processing involved the following steps: original 16S rRNA gene sequencing read demultiplexing, quality filtering with fastp version 0.20.0, and merging with Flash version 1.2.7, using the maximum mismatch rate for the overlapping region in Fast P0.20.0. Uparse 7.1 was used for clustering operational taxonomic units (OTUs) at a similarity of 97%, and chimeric sequences were identified and deleted. RDP Classifier version 2.2 was used to classify and analyze the 16S rRNA sequences; the confidence threshold was 0.7, and the classification of each representative OTU sequence was analyzed [29].

Sequencing was performed using Illumina's MiSeqPE250 and MiSeqPE300 platforms (Shanghai Majorbio Bio-pharm Technology Co., Ltd.). Raw data were uploaded to the NCBI database for comparison.

### Untargeted metabolomic assays and analysis

A 200 μL sample was accurately transferred to a 1.5 mL centrifuge tube; methanol and acetonitrile were mixed in a 1:1 ratio, and then 800 μL of this solution was added to the sample for extraction. After vortex mixing, the low-temperature ultrasonic extractor was set to 5 °C, and ultrasonic extraction was performed at 40 kHz for 30 min. After extraction was completed, the sample was placed in a freezer at -20 °C for 30 min and centrifuged at 4 °C for 15 min. After centrifugation, the supernatant was absorbed and dried with nitrogen. A mixture of acetonitrile and water at 1:1 was used as the compound solution; 120 μL was absorbed and redissolved, and then vortex mixing was performed. After low-temperature ultrasonic extraction, the sample was centrifuged at 4 °C for 10 min, and the supernatant was absorbed and transferred to an injection vial with intubation. Ultrahigh-performance liquid chromatography tandem Fourier transform mass spectrometry was performed on an UHPLC-QExactive system (Thermo Fisher Scientific, USA) system for LC‒MS detection. In addition, 20 μL of supernatant was removed from each sample and used as a quality control sample. The chromatographic conditions were as follows: the chromatography column used was an ACQUITY UPLCHSST3 (100 mm × 2.1 mm, i.d.1.8 μm; Waters, Milford, USA); mobile phase A was 95% water + 5% acetonitrile + 0.1% formic acid, and mobile phase B was 47.5% acetonitrile + 47.5% isopropanol + 5% water + 0.1% formic acid. The flow rate was set to 0.40 ml/min, the injection volume was 2 μL, and the column temperature was 40 ℃. The Majorbio cloud platform (https://cloud.majorbio.com) was used for multivariate analysis.

### Statistical analyses

The data were statistically analyzed using Excel 2019 and Statistical Product and Service Solutions (SPSS) Statistics 21, and the results are shown as the means with their standard deviations (mean ± SD). Online data analysis was performed using the free online Majorbio Cloud Platform (http://www.majorbio.com) of Majorbio Bio-Pharm Technology Co., Ltd. (Shanghai, China). Metabolic group data were analyzed using KEGG (www.kegg.jp/kegg/kegg1.html) developed by Kanehisa Laboratories [30–32].

## Results

The Ace and Chao1 indices, which describe endophytic bacterial and fungal richness, and the Shannon and Simpson indices, which describe the endophytic bacterial and fungal diversity, were not significantly different between the IS and SS sugarcane cultivars (Table 2).

**Table 2:** Endophytic bacterial and fungal diversity in insensitive (IS) and sensitive (SS) sugarcane cultivars

Treatment	Treatment	Shannon	Simpson	Ace	Chao1	Coverage
Endophytic bacteria	IS	0.93 ± 0.36a	0.70 ± 0.16a	386.90 ± 216.25a	371.08 ± 206a	0.99
	SS	1.24 ± 0.87a	0.59 ± 0.26a	340.26 ± 109.32a	326.13 ± 122.68a	1.00
Endophytic fungi	IS	2.78 ± 0.56a	0.18 ± 0.11a	156.09 ± 35.26a	160.35 ± 42.33a	1.00
	SS	2.54 ± 0.43a	0.17 ± 0.09a	141.16 ± 55.28a	142.63 ± 56.67a	1.00

Data in the table are means ± SDs. Values followed by different lowercase letters indicate significant differences between endophytic bacteria in insensitive sugarcane (IS) and sensitive sugarcane (SS) cultivars (*p* < 0.05)

**Fig. 2:**
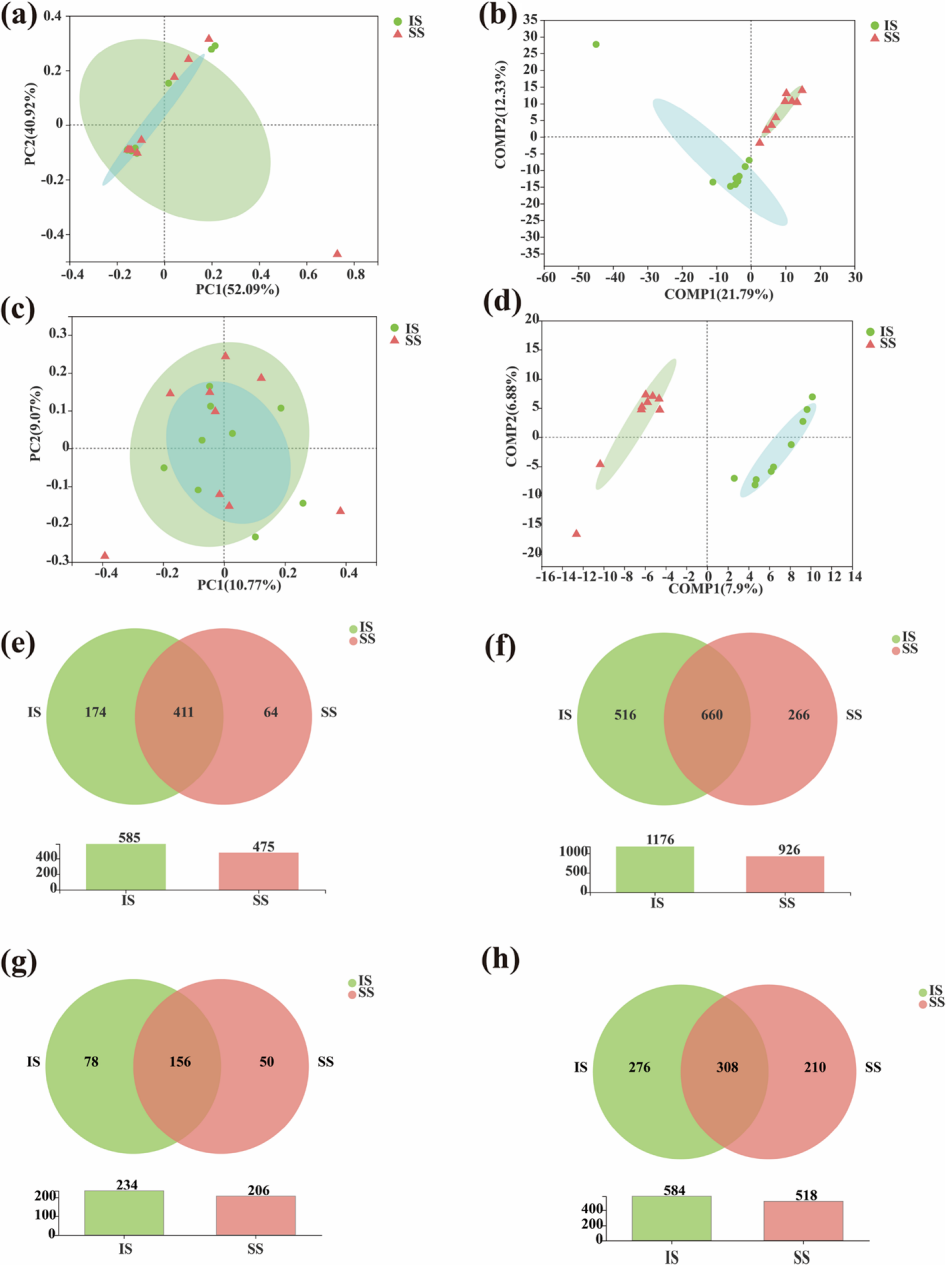
**a** PCoA of endophytic bacterial communities. **b** PLS-DA score plot of endophytic bacterial communities. **c** PCoA of endophytic fungal communities. **d** PLS-DA score plot of endophytic fungal communities. **e** Venn diagram analyses of endophytic bacteria at the genus level. **f **Venn diagram analyses of endophytic bacteria at the OTU level. **g** Venn diagram analyses of endophytic fungi at the genus level. **g** Venn diagram analyses of endophytic fungi at the OTU level. IS — insensitive sugarcane; SS — susceptible sugarcane

To evaluate the extent of the similarity of endophytic bacterial communities between insensitive and susceptible sugarcane cultivars, unweighted principal coordinate analysis (PCoA) and partial least squares discriminant analysis (PLS-DA) were performed (Fig. 2a-d). The results showed that the composition of endophytic bacteria and fungi in IS and SS sugarcane varieties was quite similar, but there were significant differences in community structure. Additionally, at the genus level and OTU level, the total number and unique number of bacteria and fungi in sugarcane stems of the IS variety were higher than those in stems of the SS variety (Fig. 2e-h).

The number of dominant endophytic bacterial phyla (i.e., relative abundances greater than 1%) in both insensitive and sensitive sugarcane cultivars was 3.

**Fig. 3:**
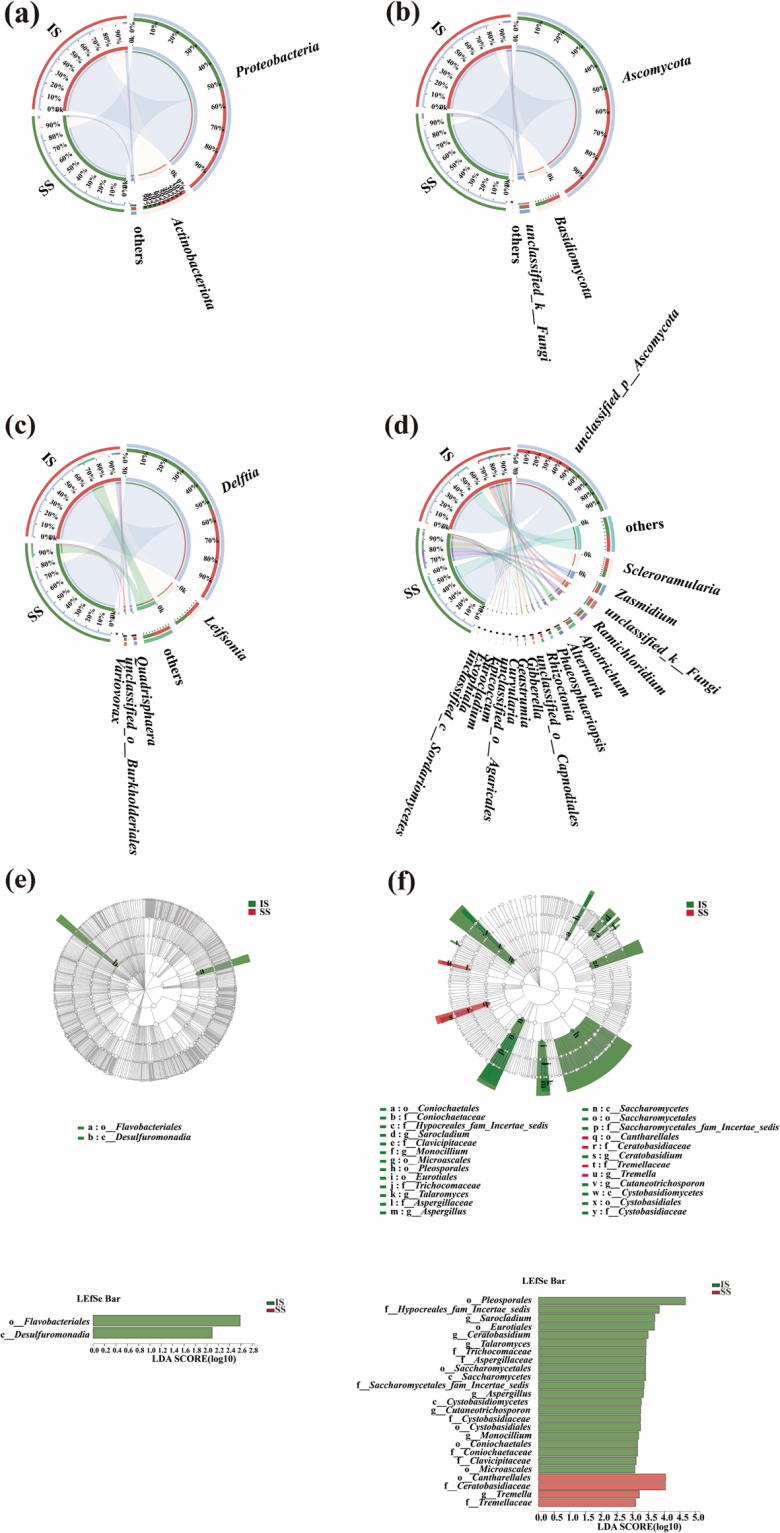
**a** Compositions of endophytic bacterial communities at the phylum level. **b** Compositions of endophytic fungal communities at the phylum level. **c** Compositions of endophytic bacterial communities at the genus level. **d** Compositions of endophytic bacterial communities at the genus level. LEfSe analysis of significantly differentially abundant bacteria (**e**) and fungi (**f**) between insensitive (IS) and sensitive (SS) sugarcane cultivars

The compositions of endophytic dominant bacteria in the stems of IS and SS cultivars at the phylum level were similar; however, their proportions were different. First, *Proteobacteria* (82.74%), *Actinobacteriota* (15.32%) and others (1.94%) were the dominant endophytic bacterial phyla of IS. In contrast, *Proteobacteria* (75.44%), *Actinobacteriota* (21.42%) and others (3.13%) were the dominant endophytic bacterial phyla of SS (Fig. 3a).

The number of dominant endophytic bacterial genera (i.e., relative abundances greater than 1%) in IS and SS was 4 and 6, respectively. *Quadrisphaera* (13.54%) and *Variovorax* (1.15%) were the unique dominant endophytic bacterial genera in the stems of SS. In contrast, IS has no unique dominant endophytic bacterial genera. Meanwhile, compared with SS, the proportions of *Delftia* (76.50%), *Leifsonia* (13.58%), and *unclassified_o__Burkholderiales* (1.75%) in IS increased markedly (Fig. 3c).

The number of dominant endophytic fungal phyla (i.e., relative abundances greater than 1%) in both insensitive and susceptible sugarcane cultivars was 3. Meanwhile, although the compositions of the dominant fungal phyla did not significantly change between IS and SS, there was a difference in relative abundance. In particular, the proportion of *Ascomycota* (86.05%) in IS increased significantly (Fig. 3b).

Furthermore, the number of dominant endophytic fungal genera (i.e., relative abundances greater than 1%) in IS and SS cultivars was 13 and 14, respectively. *Alternaria* (6.26%), *Geastrumia* (2.77%), *Sarocladium* (1.44%), *Epicoccum* (1.27%) and *Exophiala* (1.12%) were the unique dominant endophytic fungal genera in the stems of IS cultivars. In contrast, *unclassified_c__Sordariomycetes* (1.03%), *unclassified_o__Agaricales* (1.53%), *Curvularia* (1.38%), *Gibberella* (1.92%), and *unclassified_o__Capnodiales* (2.32%) were the unique dominant endophytic fungal genera in the stems of SS cultivars (Fig. 3d).

A nonparametric factorial Kruskal–Wallis (KW) rank sum test and LEfSe analysis (LDA threshold of 2) were carried out to analyze the significant differences and the main contributing biomarker classes between insensitive and susceptible sugarcane cultivars.

As shown in Fig. 3e, the compositions of the endophytic bacterial communities differed significantly in only the insensitive sugarcane cultivars. *Flavobacteriales* (order) and *Desulfuromonadia* (class) were enriched in the insensitive sugarcane cultivars only. As shown in Fig. 3f, the compositions of the endophytic fungal communities were significantly different in stems between IS and SS cultivars. For example, *Sarocladium* (genus), *Monocillium* (genus), *Talaromyces* (genus), *Aspergillaceae* (from family to genus), *Ceratobasidium* (genus), and *Cutaneotrichosporon* (genus) were enriched in the stems of IS cultivars. In contrast, *Tremella* (genus) was enriched in the stems of SS cultivars.

**Fig. 4:**
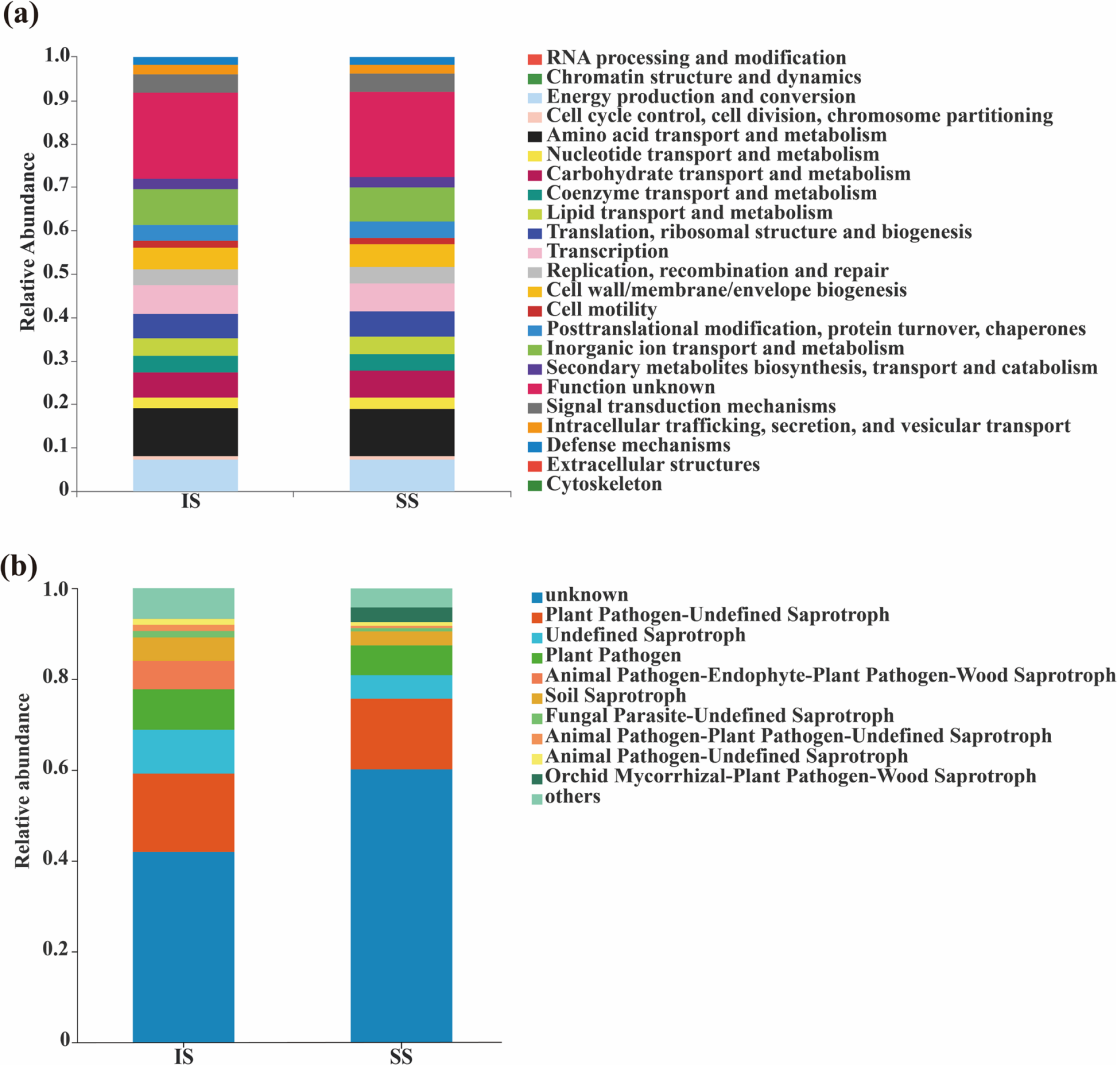
Relative abundance of Clusters of Orthologous Groups (COG) and fungal functional groups (FUNGuild) based on OTUs between insensitive (IS) and sensitive (SS) cultivars

Based on the Kyoto Encyclopedia of Genes and Genomes (KEGG) database, 23 functional types of endophytic bacteria were detected between IS and SS according to Clusters of Orthologous Groups (COG) functional classification. Although the functional types of stem endophytic bacteria were very similar between IS and SS, the 8 functional types of IS were stronger than those of SS. At the same time, the activity of 7 metabolic pathways in SS was higher than that in IS (Fig. 4a).

In addition, according to the EggNOG database, 10 unique endophytic fungal COG functions were identified between IS and SS cultivars. In comparison with SS cultivars, IS cultivars exhibited more abundant functions of endophytic fungi. For example, Animal Pathogen-Endophyte-Plant Pathogen-Wood Saprotroph (6.71%), Fungal Parasite-Undefined Saprotroph (1.55%), Animal Pathogen-Plant Pathogen-Undefined Saprotroph (1.45%), and Animal Pathogen-Undefined Saprotroph (1.39%) were the unique functional groups of IS. However, Orchid Mycorrhizal-Plant Pathogen-Wood Saprotroph (3.36%) was the only unique function of endophytic fungi in stems of SS. All of the above results indicated that the more abundant functions of endophytic fungi in the stems of IS cultivars could also be one of the reasons for their higher resistance to smut (Fig. 4b).

**Fig. 5:**
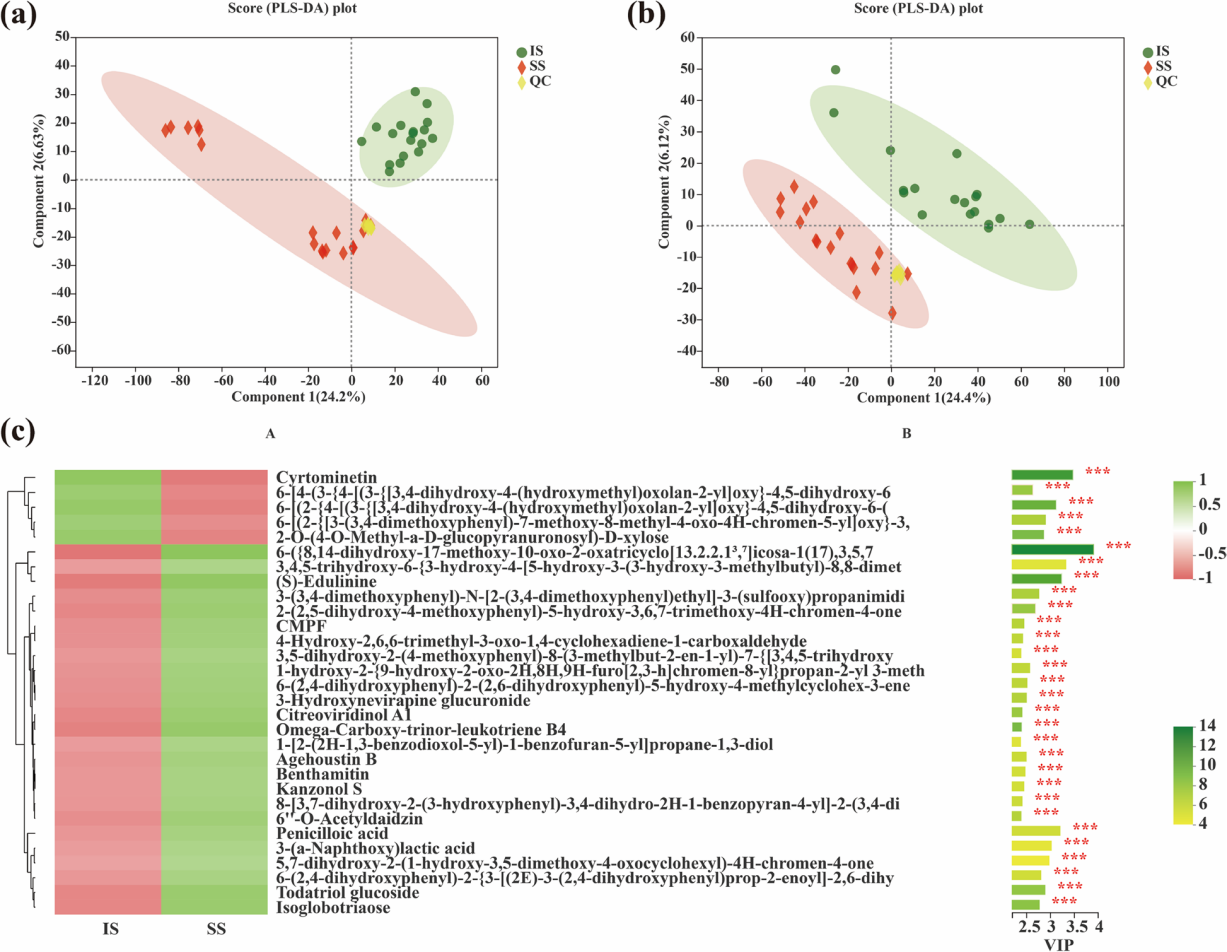
PLS-DA analysis of metabolites. **a** Liquid chromatography–mass spectrometry ESI( +); **b** liquid chromatography–mass spectrometry ESI(-). **c** Variable importance in projection (VIP) scores of metabolites in stems between IS and SS cultivars. VIP bar chart of metabolites on the right; the length of the bar indicates the contribution of this metabolite to the difference between the two groups, which is not less than 1 by default. The larger the value is, the greater the difference between the two groups. The color bar indicates the significance of differences in metabolite levels between the two groups of samples. A smaller p value is indicated by a darker color. * represents *p* < 0.05, * * represents *p* < 0.01, and * * * represents *p* < 0.001

As shown in Fig. 5 a, b, the QC samples were well grouped, indicating that the bioanalytical quality and data quality were good. There were significant differences among stem exudates.

Based on the PLS-DA model, the variable importance in projection (VIP) score described the order of the abundance of metabolites in stems between IS and SS cultivars. A higher VIP score could be considered an indicator of a higher abundance of metabolites.

For 30 most abundant metabolites, 5 metabolites were significantly upregulated and 25 metabolites were significantly downregulated is the stems of IS cultivars compared with SS cultivars. In particular, among the top five abundant metabolites, only cyrtominetin (VIP = 3.48) was significantly upregulated in the stems of IS cultivars compared to SS cultivars. In contrast, 6-({8,14-dihydroxy-17-methoxy-10-oxo-2-oxatricyclo[13.2.2.1^3^,^7^]icosa-1(17),3,5,7 (VIP = 3.92), 3,4,5-trihydroxy-6-{3-hydroxy-4-[5-hydroxy-3-(3-hydroxy-3-methylbutyl)-8,8-dimet (VIP = 3.34), (S)-edulinine (VIP = 3.25) and penicilloic acid (VIP = 3.21) were significantly downregulated in the stems of IS cultivars compared to SS cultivars (Fig. 5c).

Thirty-one amino acid metabolites were identified from IS and SS. Among them, 15 were significantly different between IS and SS. Among these metabolites, the levels of N-acetylornithine, 3-isopropylmalate, gentisic acid, 3-(3,4-dihydroxyphenyl)-2-oxopropanoic acid, 4-guanidinobutanoic acid and indole were significantly higher in the stems of IS cultivars than in those of SS. However, the levels of (S)-2-aceto-2-hydroxybutanoic acid, L-glutamic acid, N-epsilon-acetyl-L-lysine, vanillylmandelic acid, 3-(2-hydroxyphenyl) propionic acid, 3-methyl pyruvic acid, L-dopa and L-glutamine were significantly lower in the stems of IS than in those of SS.

In addition, 30 species from carbohydrate metabolism were found in the stems of the IS and SS cultivars, 15 of which were found to be significantly different between the two. The levels of alpha-lactose, 6-phosphogluconic acid, deoxyribose 5-phosphate and aconitic acid were significantly higher in the stems of IS cultivars than in those of SS. In contrast, the levels of propiolic acid, 2-phenylethanol glucuronide, N-acetylmannosamine, 3-methyl pyruvic acid, beta-D-fructose 2-phosphate, levan, myo-inositol, trehalose 6-phosphate, gluconolactone, D-glucarate and D-glucuronic acid were lower in the stems of IS cultivars than in those of SS. Moreover, 11 cofactors and vitamins were also identified in the stems of the IS and SS cultivars. Among them, the levels of niacinamide and trigonelline were significantly higher in the stems of IS than in those of SS. However, the level of maleic acid was significantly lower in the stems of IS than in those of SS (Table 3).

**Table 3:** Metabolites with significant differences between insensitive and sensitive sugarcane cultivars

Metabolite	IS/SS	Lon (M/Z)	RT (min)	*p* value	Positive/negative
Amino acid metabolism					
(S)-2-aceto-2-hydroxybutanoic acid	0.76	164.09	5.76	< 0.05	ESI +
L-glutamic acid	0.41	130.05	0.50	< 0.05	ESI +
N-epsilon-acetyl-L-lysine	0.71	189.12	1.20	< 0.05	ESI +
N-acetylornithine	1.06	157.10	8.70	< 0.05	ESI +
Vanillylmandelic acid	0.43	181.05	3.20	< 0.05	ESI +
3-(2-Hydroxyphenyl)propionic acid	0.52	167.07	3.00	< 0.05	ESI +
3-Isopropylmalate	1.71	218.10	1.40	< 0.05	ESI +
4-Guanidinobutanoic acid	2.78	146.09	1.21	< 0.05	ESI +
3-Methyl pyruvic acid	0.71	103.04	0.91	< 0.05	ESI +
L-dopa	0.47	458.15	0.83	< 0.05	ESI +
Indole	1.16	118.07	3.45	< 0.05	ESI +
L-glutamine	0.48	145.06	0.72	< 0.05	ESI-
Gentisic acid	1.09	153.02	2.99	< 0.05	ESI-
3-(3,4-Dihydroxyphenyl)-2-oxopropanoic acid	4.53	437.08	2.78	< 0.05	ESI-
Carbohydrate metabolism					
Alpha-lactose	1.11	360.15	0.88	< 0.05	ESI +
6-Phosphogluconic acid	1.89	314.99	0.52	< 0.05	ESI +
Propiolic acid	0.76	71.01	0.87	< 0.05	ESI +
2-Phenylethanol glucuronide	0.22	619.20	5.04	< 0.05	ESI +
N-acetylmannosamine	0.64	186.08	2.39	< 0.05	ESI +
3-Methyl pyruvic acid	0.67	103.04	0.91	< 0.05	ESI +
Beta-D-Fructose 2-phosphate	0.10	261.04	0.81	< 0.05	ESI +
Levan	0.96	522.20	0.57	< 0.05	ESI +
Deoxyribose 5-phosphate	2.17	250.97	0.72	< 0.05	ESI-
Myo-inositol	0.84	179.06	0.87	< 0.05	ESI-
Trehalose 6-phosphate	0.21	421.07	0.76	< 0.05	ESI-
Gluconolactone	0.58	223.05	0.53	< 0.05	ESI-
D-glucarate	0.42	209.03	0.52	< 0.05	ESI-
Aconitic acid	1.28	173.01	2.36	< 0.05	ESI-
D-glucuronic acid	0.71	193.03	0.53	< 0.05	ESI-
Metabolism of other amino acids					
Propiolic acid	0.81	71.01	0.87	< 0.05	ESI +
L-tyrosine	0.93	180.07	2.10	< 0.05	ESI-
Metabolism of cofactors and vitamins					
Niacinamide	2.56	123.06	1.10	< 0.05	ESI +
Trigonelline	3.37	176.01	0.67	< 0.05	ESI +
Maleic acid	0.64	115.007	0.50	< 0.05	ESI-

*IS* insensitive sugarcane, *SS* sensitive sugarcane, *RT* retention time, *M/Z* an experimentally observed value. ESI + , positive ion mode; ESI − , negative ion mode

**Fig. 6:**
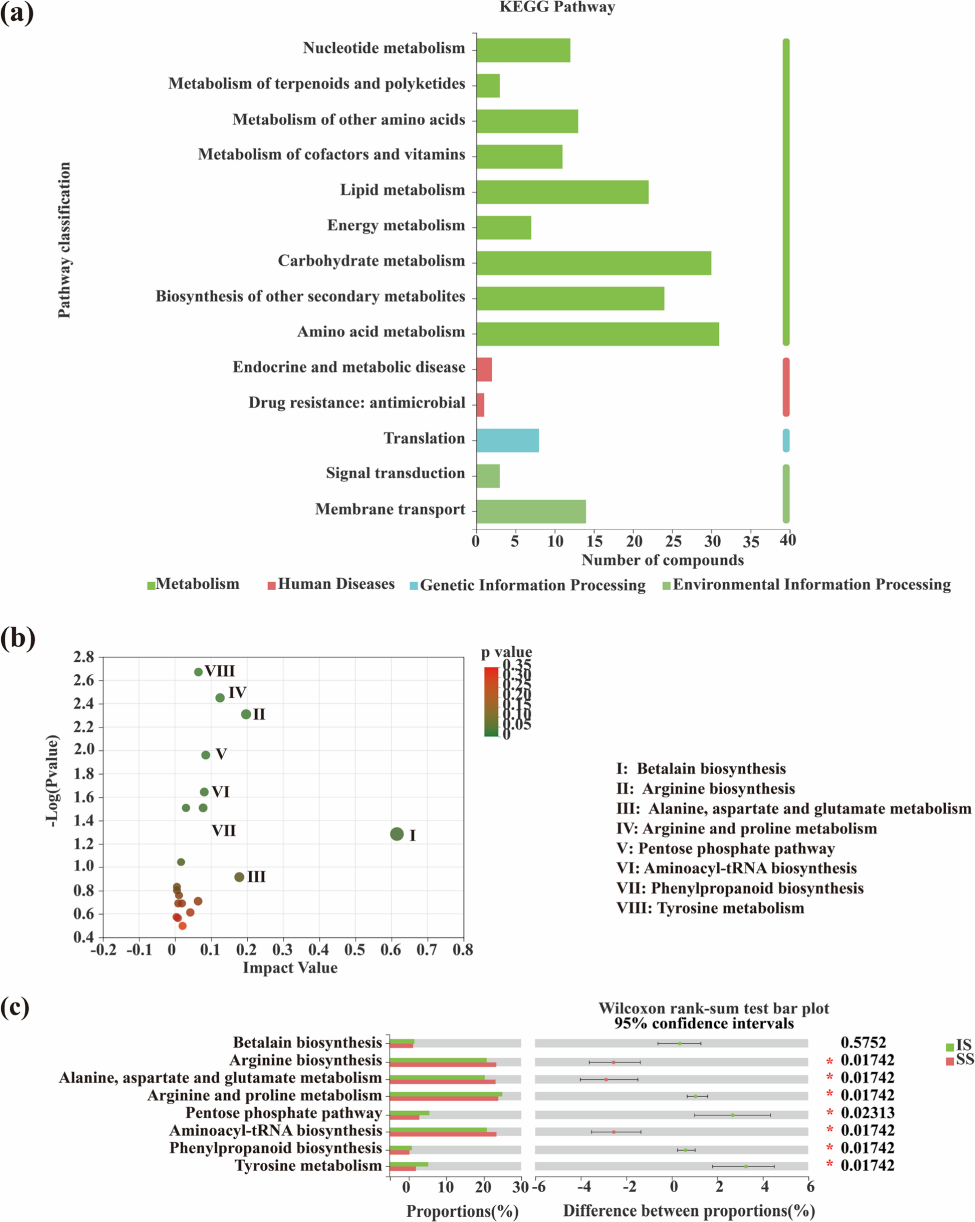
**a** KEGG metabolic pathways of metabolites in stems of IS and SS cultivars. **b** Metabolic pathway enrichment analysis of differentially abundant metabolites. **c** Changes in metabolites in different metabolic pathways by KEGG enrichment analysis between insensitive (IS) and sensitive (SS) sugarcane cultivars

In addition, based on the KEGG database, all metabolites derived from two smut-resistant sugarcane cultivars were classified into 14 secondary metabolic pathways. In the primary metabolic pathway, 122 metabolites were classified under Metabolism; 16 metabolites were classified under Environmental Information Processing; 8 metabolites were classified under Genetic Information Processing; and 3 metabolites were classified under Human Diseases (Fig. 6a).

Topological analysis was carried out according to the number of metabolites contained in the metabolic pathway. The *p* value was low and pathway influence factor was high between insensitive sugarcane and sensitive sugarcane, indicating that these 8 pathways changed markedly. Among these metabolic pathways, Arginine and proline metabolism, Pentose phosphate pathway, Phenylpropanoid biosynthesis, Tyrosine metabolism were significantly more abundant in insensitive sugarcane than in sensitive sugarcane. However, alanine, aspartate and glutamate metabolism, arginine biosynthesis and aminoacyl-tRNA biosynthesis were significantly less abundant than in insensitive sugarcane (Fig. 6b, c).

The correlation between endophytic microorganisms and the 20 most abundant metabolites was calculated and analyzed by using the Spearman correlation algorithm and Bray–Curtis distance algorithm.

The fungi of *Apiotrichum*, *Zasmidium* and *Ramichloridium* were the common dominant fungi in stems of the IS and SS cultivars, among which *Apiotrichum* and *Zasmidium* were significantly negatively correlated with ethyl syringate; *Ramichloridium* was significantly negatively correlated with the metabolites 1-linoleoylglycerophosphocholine, LysoPE(18:2(9Z,12Z)/0:0) and PE(18:2/0:0).

The fungi of *Exophiala* and *Sarocladium* were the unique dominant fungi of IS, between which *Exophiala* was significantly positively correlated with the metabolite 3,4,5-trihydroxy-6-[(3-phenylpropanoyl)oxy]oxane-2-carboxylic acid. *Sarocladium* was significantly negatively correlated with the metabolite L-2-amino-5-(methylthio)pentanoic acid.

The fungi of *unclassified_c__Sordariomycetes* and *Gibberella* were the unique dominant fungi of SS, between which *unclassified_c__Sordariomycetes* was significantly positively correlated with the metabolite Ramiprilat and negatively correlated with the metabolite 2-benzylpropanedioic acid.

**Fig. 7:**
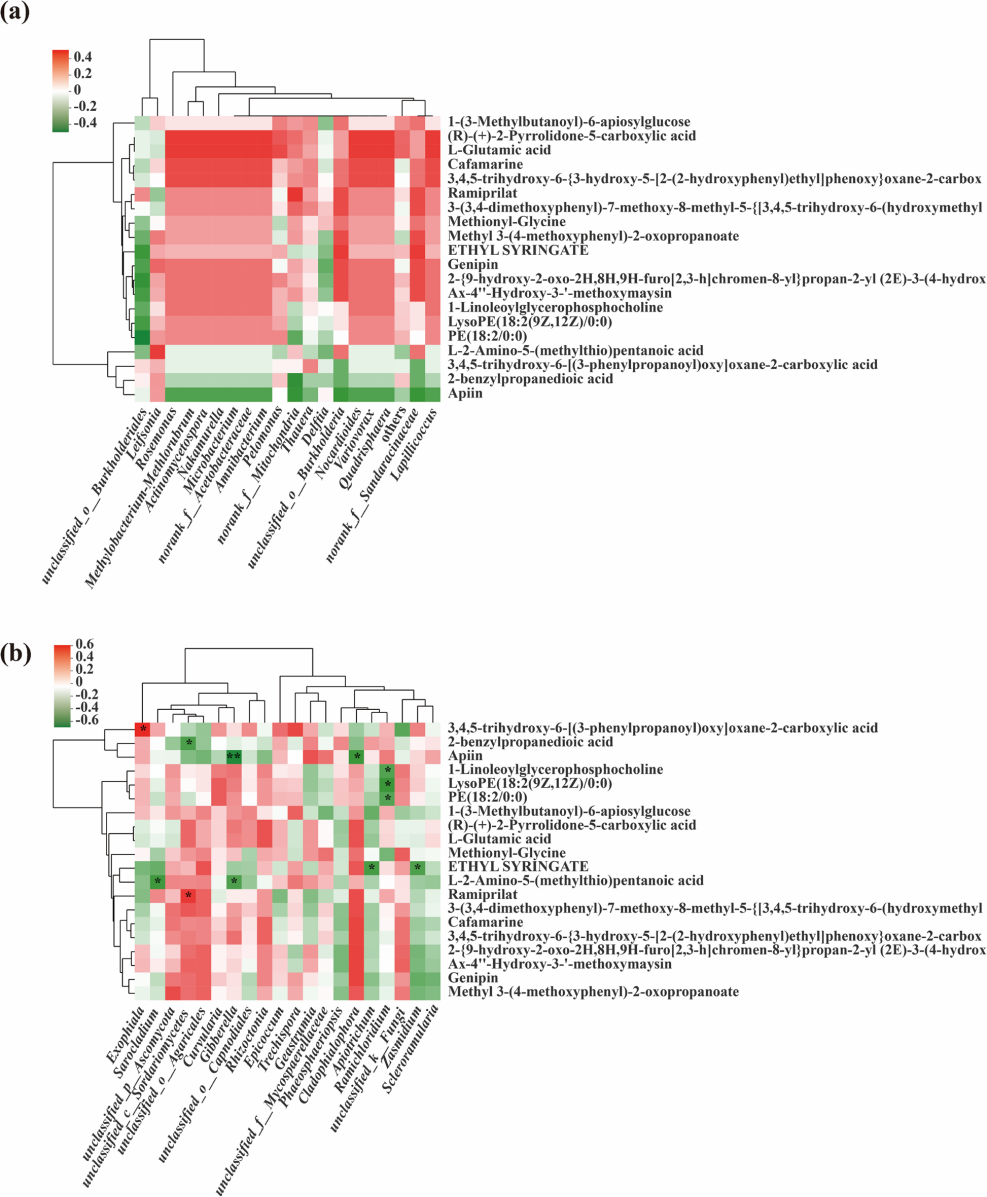
Correlation between metabolites and bacterial (**a**) and fungal (**b**) communities between IS and SS cultivars Heatmaps were used to analyze the Spearman correlation coefficient (rho value) and *P* value of metabolites and flora. Significant differences are indicated as follows: *0.01 < *p* < 0.05, **0.001 < *p* < 0.01, *** *p* < 0.001. The clustering at the top and left of the graph shows the metabolites of bacterial groups and the results of hierarchical clustering based on Euclidean distance, respectively

The fungi of *Gibberella* were significantly negatively correlated with the metabolites L-2-amino-5-(methylthio)pentanoic acid, *Cladophialophora*, and Apiin (Fig. 7a).

In summary, metabolomics found that there were a large number of metabolites in the stems of IS and SS, and various metabolites were correlated with the endophytic fungal flora in the stems but were not related to the endophytic bacteria (Fig. 7b).

## Discussion

*S. scitamineum* infection could cause a strong nonspecific defense response in sugarcane and leads to significant changes in the transcription of plant hormone gene signals [9, 33]. Resistant varieties are characterized by oxidative bursts, early tissue lignification and upregulation of chitinase and disease resistance genes. The early changes in the meristem and the late upregulation of the lignin pathway in susceptible varieties easily lead to whiplash development [3]. As the chemical substances in traditional fungicides have difficulty penetrating the waxy layer of sugarcane stems, they have difficulty reaching the inner part of sugarcane stems to exert effects [34]. In contrast, biological control instead of traditional chemical control could overcome the limitations of the effectiveness of chemical fungicides against sugarcane smut [35].

Meanwhile, *S. scitamineum* infection affects sugarcane resistance-related metabolic pathways, such as plant‒pathogen interaction, plant hormone signal transduction, phenylalanine metabolism, peroxisome, flavonoid biosynthesis, phenylpropane biosynthesis and ribosome. Moreover, in susceptible plants, a slow response and weak defense signals induce the spread of pathogens throughout the plant and damage the plant. The main difference between resistant and susceptible plants is how long the host can recognize pathogen invasion and respond to invasion [36–40]*.*

Endophytic microorganisms are ubiquitous in plants and can promote plant growth and enhance plant stress resistance [41, 42]. The disease resistance of plants is an important factor driving changes in endophytic microbial communities [43, 44]. Endophytic microorganisms can induce plant self-defense mechanisms, reduce pathogen deposition and increase plant growth and adaptability [45, 46]. The self-defense function of plants can also be realized by chemicals produced by endophytic fungi [47].

Endophytes play an active role in plant disease resistance and growth. In our experiment, we found that *Leifsonia* and *unclassified_o_Burkholderiales* were the dominant bacterial genera, and *Ramichloridium* was the dominant fungal genus, of IS cultivars; their abundances were all higher in IS cultivars than in SS cultivars. Meanwhile, *Alternaria*, *Sarocladium*, *Epicoccum* and *Exophiala* were the dominant fungal genera of IS. Moreover, based on the LEfSe analysis, a significant contribution of *Talaromyces* in stems of IS varieties was also observed. Many studies have proven that *Leifsonia* species possess bacteriostatic activity [48, 49] and can encode anti-host reactive oxygen species, such as iron peroxidase and arginase [50]. *Burkholderiales* species have strong inhibitory activity against the sugarcane smut pathogen [35]. Additionally, endophytic fungi, such as *Sarocladium* [51], *Epicoccum* [52, 53], *Exophiala* [54, 55], *Apiotrichum* [56], and *Phaeosphaeriopsis* [57] species, can protect hosts from pathogen attacks. Furthermore, *Talaromyces* can enhance plant resistance [58], and *Ramichloridium* can promote plant growth [59]. Additionally, salicylic acid [60, 61], ethylene [62] and jasmonic acid [61] all participate in signaling pathways related to sugarcane smut infection. Resistant cultivars were shown to contain more salicylic acid [63], jasmonic acid [37] and ethylene [64] than sensitive cultivars. *Alternaria* can induce ethylene release [65] and increase salicylic acid content [66], while *Sarocladium* can promote jasmonic acid accumulation [67]. These findings are all consistent with our results.

In addition, PICRUSt functional prediction showed that among secondary metabolic pathways, the copy number of multifunctional genes in IS was significantly lower than that in SS. Moreover, the endophytic bacteria of SS had stronger signaling molecules and interactions. Furthermore, FUNGuild results also showed that Animal Pathogen-Endophyte-Plant Pathogen-Wood Saprotroph was the unique functional type of endophytic fungi in stems of IS. Therefore, the enrichment of resistant fungi and bacteria and higher levels of ethylene, salicylic acid and jasmonic acid in stems of smut-insensitive sugarcane cultivars are important mechanisms underlying their anti-smut properties.

Plant resistance is also related to metabolic pathways. For example, indole had an obvious effect on smut [68]; niacinamide also had certain antibacterial activity and was enriched in smut-resistant varieties [69]; gentisic acid had antibacterial activity [70]; and arginine had a significant positive effect on smut [71] and could be synthesized from N-acetylornithine [72].

Additionally, our results also showed that metabolic pathways, such as the N-acetyl ornithine, gentisic acid, indole, aconitic acid and niacinamide pathways, were all significantly enriched in IS compared with SS cultivars. Meanwhile, the levels of glutamic acid increased and those of methionine decreased in the stems of IS compared with SS cultivars. The abundances of arginine and proline metabolism, the pentose phosphate pathway, phenylpropanoid biosynthesis and tyrosine metabolism in smut-insensitive were significantly higher than those in SS. In contrast, the abundance of alanine, aspartate and glutamate metabolism was significantly lower in IS cultivars than in SS cultivars. This was similar to the results of previous studies. Furthermore, proline is an important substance in the regulation of plant physiology; it can improve cell detoxification activity and protect plants from biological stress [68, 73]. Arginine and proline metabolism is beneficial to plant signal transduction and regulation under stress and can improve plant adaptability to the environment [74]. The methionine and pentose phosphate pathways also play an important role in antioxidant metabolism [75]. Tyrosine is the starting point of phenylpropane biosynthesis in some fungi and bacteria [76, 77]. Additionally, phenylpropanoid biosynthesis is essential for plant survival, improving plant resistance and tolerance to biotic and abiotic stress and protecting plants from injury [78, 79].

Our results also showed that metabolic pathways, such as the N-acetyl ornithine, gentisic acid, indole, acoustical acid and niacinamide pathways, were all significantly enriched in IS compared with SS cultivars. Meanwhile, the levels of glutamic acid increased and the levels of methionine decreased in the stems of IS compared with those of SS cultivars. In addition, the abundances of arginine and proline metabolism, the pentose phosphate pathway, phenylpropanoid biosynthesis and tyrosine metabolism in the smut-insensitive sugarcane cultivars were significantly higher than those in SS. However, in contrast, the abundance of alanine, aspartate and glutamate metabolism in IS cultivars was significantly lower than that in SS. Moreover, some metabolites, such as flavonoids, are inhibitors of teliospore germination [80], and Apiin is a flavonoid [81]. *Gibberella*, one of the dominant fungal genera enriched in stems of sugarcane cultivars susceptible to smut, was significantly negatively correlated with Apiin. All of the above results indicated that the different metabolomic profiles in stems between smut-insensitive and smut-susceptible sugarcane cultivars are important reasons for their different responses to smut.

## Conclusions

Higher abundances of endophytic bacteria and fungi, which produce ethylene, salicylic acid and jasmonic acid; the enrichment of fungi with anti-smut activity, such as *Ramichloridium*, *Alternaria*, *Sarocladium*, *Epicoccum* and *Exophiala;* and higher activities of the pentose phosphate pathway, phenylpropanoid biosynthesis, arginine and proline metabolism, and tyrosine metabolism in stems of smut-insensitive sugarcane cultivars are all speculated to be important reasons for the higher smut resistance of insensitive sugarcane cultivars. In contrast, *Gibberella*, a unique dominant fungal genus and one with strong alanine and aspartate and glutamate metabolism, present in stems of smut-susceptible sugarcane cultivars is also an important cause for the relatively low resistance to smut of these cultivars.

## Data Availability

Raw data for endophytic bacterial and fungal sequencing were deposited in the NCBI Sequence Read Archive (SRA) database under accession numbers PRJNA928696 and PRJNA928707, respectively.
